# Maropitant Citrate Administration Significantly Decreases the Rate of Peristalsis in the Stomach and Jejunum and Does Not Significantly Alter Intestinal Diameter or Intestinal Wall Thickness in Healthy Adult Dogs

**DOI:** 10.1111/vru.70167

**Published:** 2026-05-04

**Authors:** Jillian Myers, Andra Voges, Robert Werner, Nicola Ritter

**Affiliations:** ^1^ Large Animal Clinical Sciences Texas A&M University College Station Texas USA; ^2^ VitalRads Cypress Texas USA; ^3^ School of Veterinary Medicine and Biomedical Sciences Texas A&M University College Station Texas USA

**Keywords:** gastrointestinal motility, ileus, neurokinin‐1, substance P

## Abstract

Maropitant citrate (Cerenia) is a neurokinin‐1 (NK‐1) receptor antagonist labeled for the treatment of vomiting in dogs and cats. Anecdotal observation at the authors’ institution suggests that patients receiving maropitant citrate occasionally display sonographic and radiographic signs of diffuse functional ileus. The goals of this prospective one‐group pretest, posttest study were to determine if administration of maropitant citrate caused changes in ultrasonographic wall thickness and peristaltic rate at the pylorus, duodenum, or jejunum and/or a radiographic change in intestinal diameter or appearance. Twenty‐one healthy adult dogs (ages 1–8 years) were recruited. After overnight fasting, abdominal radiographs and a brief gastrointestinal ultrasound were performed at time 0 (baseline), followed by a 1 mg/kg subcutaneous dose of maropitant citrate. Imaging was then repeated 1 and 3 h post maropitant administration. Peristatic contractions and wall thickness at the pylorus, duodenum, and jejunum were measured with ultrasound, and radiographs were assessed for signs of functional ileus (mild diffuse dilation of the small intestine or mild increase in overall small intestinal gas content). Significant decrease in rate of peristalsis of the pylorus (*p* < 0.001, *hp* ^2^ = 0.48) between 0 and 1 h and between 0 and 3 h and jejunum (*p* < 0.02, *hp* ^2^ = 0.48) between 0 and 1 h was found after administration of maropitant citrate. No significant change in duodenal peristalsis or radiographic small intestinal gas or fluid distention or overall gas content.

**Abbreviations**
NK‐1: neurokinin‐1

## 1 Introduction

Diagnosis of complete small intestinal mechanical obstruction relies on radiographic or ultrasonographic identification of gastrointestinal foreign material or segmental distention of the small intestinal tract. In a complete mechanical obstruction, increased motility and secretory activity of the intestine orad to the obstruction and decreased motor activity aborad lead to segmental distention and the characteristic imaging appearance of two populations of bowel [1, 2]. Increased secretory activity of the orad segment is mediated by substance P and neurokinin‐1 (NK‐1) receptors in the intestine [3]. Mechanical obstruction also leads to vomiting mediated by the chemoreceptor trigger zone (CRTZ) and the emetic center [4, 5]. Maropitant citrate (Cerenia^1^) is an antiemetic drug that blocks the action of substance P at NK‐1 receptors [6, 7]. In the normal GI tract, substance P and NK‐1 are involved in smooth muscle contractility due to their expression on interstitial cells of Cajal, which are responsible for creating action potentials stimulating peristalsis and contractions [8, 9, 10, 11]. Maropitant citrate is well tolerated in dogs, with only a few side effects and contraindications [6, 12]. The most common side effects include transient pain at the injection site, mild lethargy, and diarrhea.Maropitant is contraindicated in dogs with significant liver dysfunction [6].

Anecdotal observations at the authors’ institutions suggest that patients receiving maropitant citrate as part of their treatment plan display ultrasonographically reduced gastrointestinal peristalsis and radiographic signs of a diffuse functional ileus, which may fail to display the typical two populations of small intestine and segmental small intestinal distention, even in the face of complete mechanical obstruction. This complication may prolong the time to diagnosis and treatment and may adversely affect patient outcome in these cases.

Maropitant administration in rabbits has been associated with increased fecal output, presumably secondary to effects specifically in the distal colon in this species [13]. A literature search of PubMed, Wiley, and ScienceDirect did not yield any studies on the effects of maropitant citrate on gastrointestinal motility or peristalsis, or on its effects on gastrointestinal imaging studies. The effects of maropitant citrate on gastrointestinal peristalsis and the ultrasonographic and radiographic appearance of the stomach and small intestine of dogs have not been previously studied. One abstract is available examining the effect of maropitant citrate on gastrointestinal motility in dogs, which concluded that maropitant citrate may decrease contraction pressure in the GI tract without altering transit time [14]. No studies could be found examining its effects on the gastrointestinal wall layering and thickness. Given that administration of maropitant citrate has become ubiquitous in both hospitalized and outpatient treatment plans for a variety of conditions, investigating and understanding the potential effects of this drug is necessary when evaluating diagnostic imaging studies in these patients.

The goals of this study were to determine if administration of maropitant citrate caused radiographic changes in small intestinal diameter and/or ultrasonographic changes in total wall thickness, individual wall layer thickness (mucosa, muscularis, or submucosa), or peristaltic rate at the pylorus, duodenum, and/or jejunum in dogs. The authors hypothesized that administration of maropitant citrate to dogs would cause diffuse distention of the small intestine, mild intestinal wall thinning, and decreased rate of peristalsis in the stomach, duodenum, and jejunum.

## 2 Materials and Methods

### 2.1 Case Selection

This was a single‐center, prospective, one‐group pretest, posttest study. Twenty‐one healthy dogs owned by the faculty, staff, and students of Texas A&M Small Animal Teaching Hospital were voluntarily recruited for this study, which was approved by the Institutional Animal Care and Use Committee. Sample size was determined by a power analysis prior to patient recruitment. Adult dogs were eligible for enrollment if they were between 1 and 8 years of age, 5–40 kg, had a body condition score of 3–7 of 9, were not pregnant or lactating, could be safely fasted for at least 8 h, and were amenable to physical restraint for radiographs and a brief abdominal ultrasound without significant stress or the need for sedation. Dogs were not excluded for having a previously diagnosed chronic enteropathy or previous gastrointestinal surgery as long as they were free of signs of gastrointestinal disease for the prior 14 days (anorexia, vomiting, and diarrhea). Dogs underwent a routine physical examination prior to enrollment. Age, breed, sex, weight in kg, current medications, diet, and time of last meal were recorded for each patient.

### 2.2 Data Recording

After a fasting period of at least 8 h, baseline imaging was obtained, consisting of right lateral, left lateral, and ventrodorsal abdominal radiographs,^2^ followed by a brief gastrointestinal ultrasound (Samsung RS80A, Samsung RS85)^3^ performed by a second‐year diagnostic imaging resident (JM). For ultrasonographic examination, animals were positioned in left lateral and/or dorsal recumbency. The transducer, which allowed for the best image resolution for each individual dog, was utilized (CA1‐7 MHz, CA4‐10 MHz, and LA4‐18 MHz). No sedation was administered for the imaging examinations. Using ultrasound, the pylorus, descending duodenum, and a segment of jejunum were observed in a sagittal plane for three minutes, and the number of propulsive contractions at each site was recorded as previously published [15]. Three separate measurements of pyloric and intestinal wall thickness for each area were taken on the frozen static image (recorded in millimeters to the hundredth digit).

Following baseline imaging, each patient was subcutaneously administered a 1 mg/kg dose of maropitant citrate. One hour following administration, abdominal studies of both imaging modalities were repeated, and 3 h post administration, ultrasound only was repeated. Between imaging studies, animals were housed in hospital kennels and provided free access to water.

Radiographic and ultrasound DICOM images were stored in the hospital PACS (Mach 7^4^) and reviewed on a commercial reviewing software (eUnity 7.0). Measurements of individual ultrasonographic gastric and small intestinal wall layers (mucosa, submucosa, and muscularis) and overall wall thickness were obtained by a second‐year resident (JM) utilizing the viewing software.

Radiographs were assessed in two rounds. First, anonymized and randomized studies were evaluated by two ACVR board‐certified radiologists (A.V., R.W.) who were blinded to the treatment status of the patient (pre‐ or post‐maropitant citrate administration). Subjective overall percentage of gas content of the small intestinal tract was estimated into one of four categories (<25%, 26%–50%, 51%–75%, or >75%), and estimated (no calipers or other measuring tool provided) ratio of largest small intestinal diameter to the height of the L5 vertebral body (≤1×, 1–1.5×, or >1.5×) was recorded. Subsequently, anonymization was removed, and the same reviewers again subjectively compared the overall small intestinal gas content and degree of distention (jejunal:L5 ratio) between pre‐ and post‐studies for each dog and classified changes for each as increased, decreased, or no change. This subsequent assessment was performed to mimic a clinical scenario in which a reviewer would have access to initial and follow‐up radiographs for a patient. Lastly, a second‐year diagnostic imaging resident (JM) measured with calipers and recorded the SI:L5 ratio of pre‐ and post‐radiographic studies by identifying the largest small intestinal segment on lateral projections (in millimeters) on randomized and anonymized radiographs, as previously described [16, 17]. This last analysis was performed so that objective measurements could be assessed in addition to the subjective assessment.

### 2.3 Statistical Analysis

Statistical analysis was performed using SPSS version 28.0.^5^ Statistical significance threshold was set to *p* < 0.05. For the dataset, residuals from the repeated measures mixed model were normally distributed (Shapiro–Wilk statistic = 0.995, *p* = 0.759), indicating that the assumption of normality was satisfied for the repeated measures analysis. To assess the rate of peristalsis, overall wall thickness, and individual wall layer thickness at the three segments between time points, repeated measures ANOVA was performed. Where a significant difference was found, a post hoc Bonferroni test was performed to determine between which time points the difference was found. The same analysis was repeated for the peristalsis data after excluding patients with no contractions at time 0 (baseline) to better assess if motility decreased in patients who exhibited any peristalsis at baseline. The Pearson correlation coefficient was calculated to determine if the length of the fasting period affected the baseline contractions observed at each site.

Intraclass correlation coefficients were performed for both subjective assessments of the volume of small intestinal gas and small intestinal distension level to determine the level of interrater reliability.

A paired *t*‐test was performed to assess the change in the small intestinal diameter to L5 ratios before and after treatment with maropitant.

## 3 Results

### 3.1 Patients

Twenty‐one dogs were enrolled in this study. The median age was 5 years old (range 1.5–7.5 years). The average weight was 21.3 kg (range 5.7–42.2 kg). Breeds included mixed breed dogs (*n* = 10), Rhodesian Ridgeback (*n* = 3), Labrador Retriever (*N* = 2), and one each of: Miniature Dachshund, Standard Dachshund, Golden Retriever, Pembroke Welsh Corgi, Boxer, and West Highland White Terrier. There were 11 males (9 castrated, 2 intact) and 10 females (9 spayed, 1 intact). The average fasting period to the time of baseline imaging was 13.9 h (range 11.5–15.6 h).

### 3.2 Rate of Peristalsis

**FIGURE 1:**
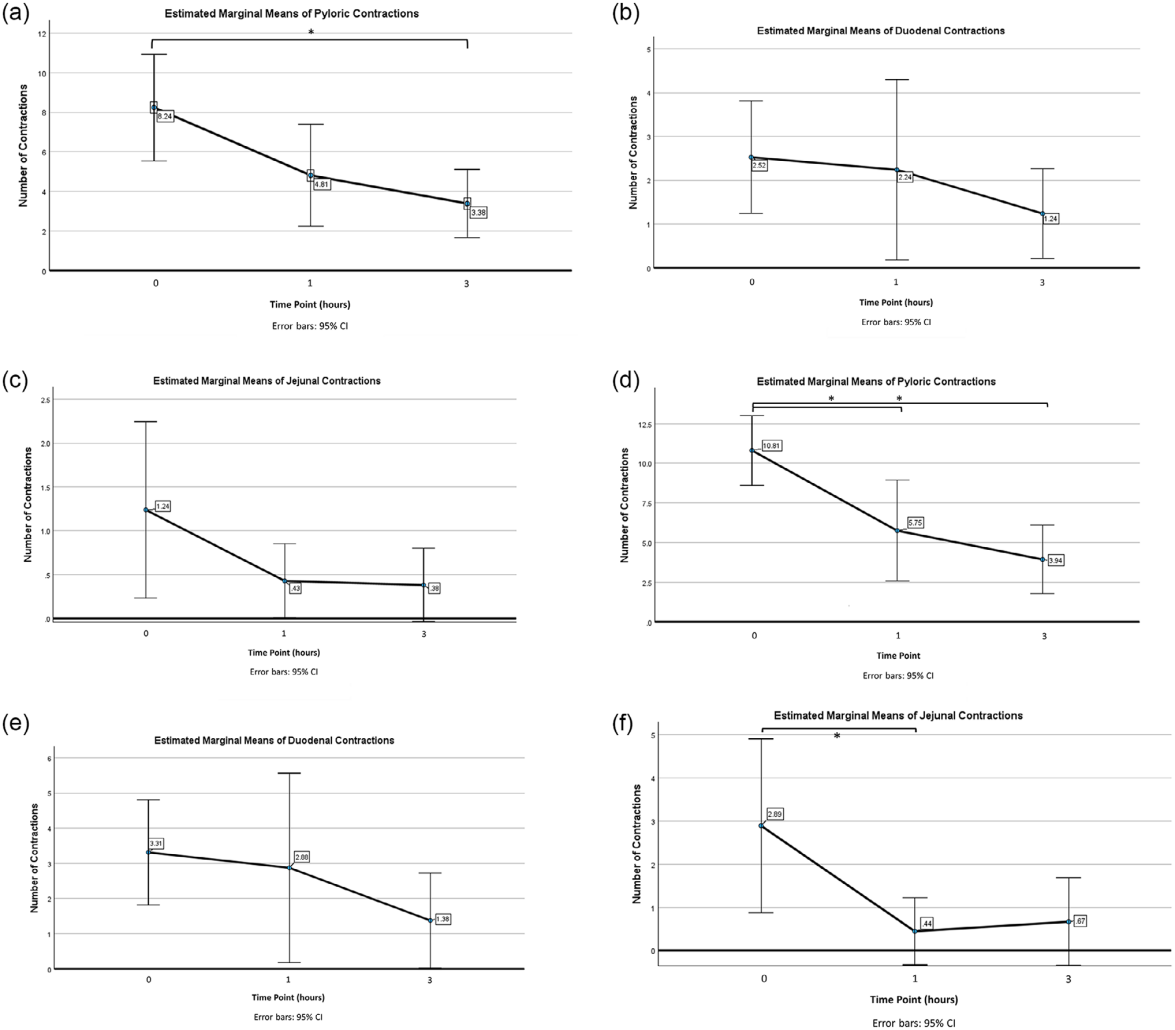
Mean peristaltic contractions at each site (pylorus, duodenum, and jejunum) and time point (baseline, 1‐ and 3‐h post maropitant administration). Mean peristaltic contractions observed at the pylorus, duodenum, and jejunum, including patients with baseline contractions of 0 (a–c) and with those patients excluded (d–f). (Bold indicates statistical significance).

Peristaltic contractions at each site are summarized in Table 1. Pyloric contractions were significantly decreased between 0 and 3 h (*n* = 21, *p* = 0.001, *ƞp* ^2^ = 0.29). When excluding patients with baseline contractions of 0, the level of significance increased (*n* = 16, *p* < 0.001, *ƞp* ^2^ = 0.48), and significant decreases were found between both 0 and 1 h (*p* = 0.01) and 0 and 3 h (*p* < 0.001). Duodenal contraction differences were not statistically significant across three time points when all patients were included (*n* = 21, *p* = 0.315), nor were significant differences found after excluding patients with baseline contractions of 0 (*n* = 16, *p* = 0.220). Jejunal contraction number differences were not statistically significant across the three time points (*n* = 21, *p* = 0.073) when all patients were included, but a significant decrease was found after excluding patients with 0 baseline contractions (*n* = 9, *p* = 0.005, *ƞp* ^2^ = 0.48), with the difference found between time points 0 and 1 h (*p* < 0.02) (Figure 1a–f). No correlation was found between the length of the fasting period and baseline contractions at each site: pylorus *r *= −0.075 (*p* = 0.759), duodenum *r* = 0.06 (*p* = 0.805), and jejunum *r* = −0.272 (*p* = 0.259).

**TABLE 1:** Summarized *p* values for number of contractions at each site and time point, with and without patients with baseline of 0 contractions included.

Contractions	Baseline 0 included	Baseline 0 excluded
Pylorus	***p* = 0.001**	***p* < 0.001**
Duodenum	*p* = 0.135	*p* = 0.220
Jejunum	*p* = 0.073	***p* < 0.005**

### 3.3 Wall Thickness

**FIGURE 2:**
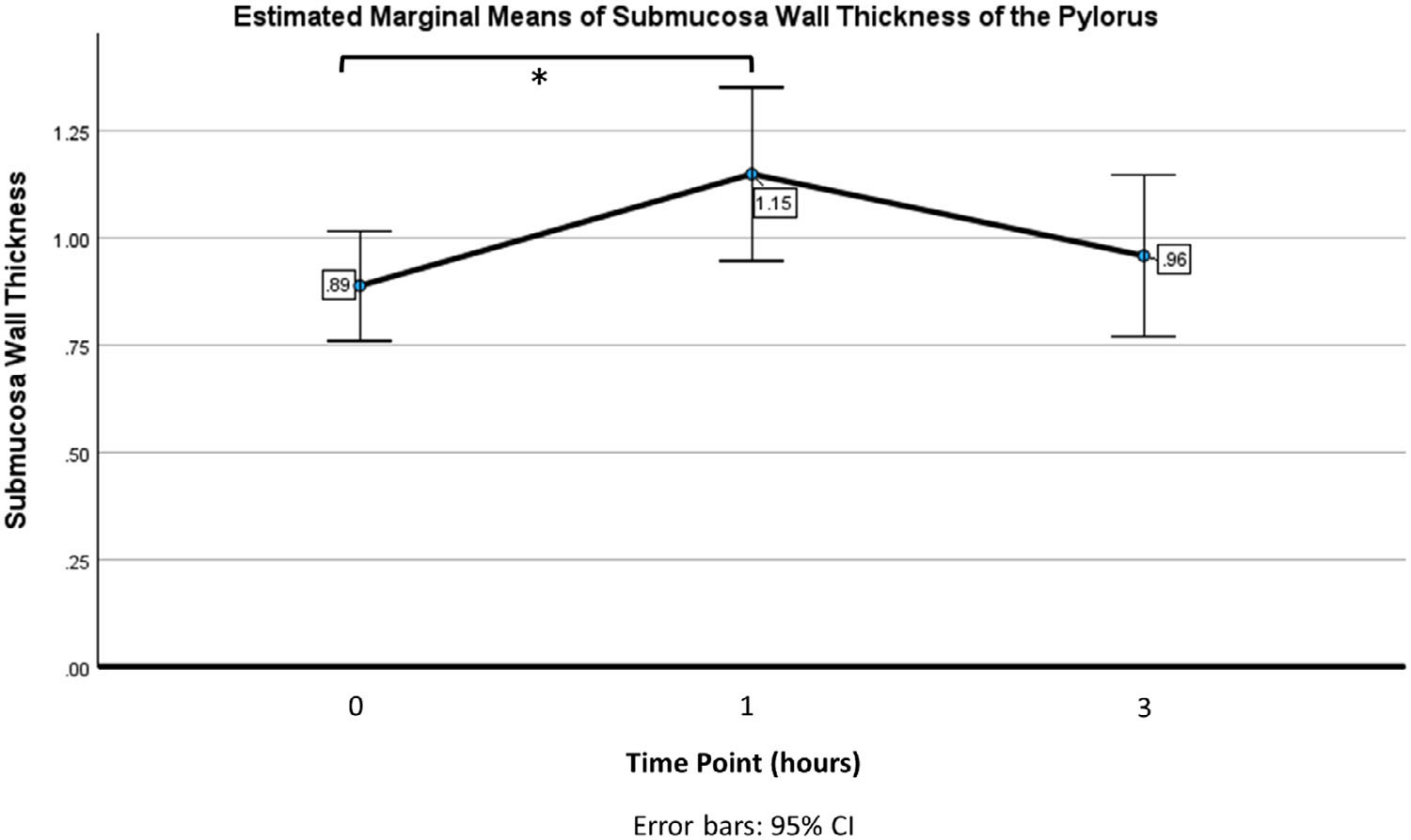
The mean submucosal thickness at the pylorus increased between baseline and 1 h post maropitant administration. (*Indicates statistical significance between values).

The overall wall thickness of the pylorus, duodenum, and jejunum was not statistically significant across three time points (*p* = 0.06, *p* = 0.68, *p* = 0.72, respectively). The only statistical difference in individual wall layer thickness was found at the pyloric submucosa (*p* = 0.03, *ƞp* ^2^ = 0.23), with a significant increase present between time 0 and 1 h (*p* < 0.04) (Figure 2). This finding, although statistically significant, likely has no clinical significance.

### 3.4 Small Intestinal Gas Content and Diameter

For the subjective gas‐distention grades, reviewer one predominantly scored “no change,” and reviewer two predominantly scored “decrease” in the subjective overall gas distention of the small intestine after maropitant administration. When assessing overall gas distention of the small intestine, interrater reliability was “good” both before (ICC = 0.732, *p *< 0.001) and after maropitant administration (ICC = 0.754, *p *< 0.001). However, the reliability of the rater differences was poor (ICC = 0.159, *p *= 0.490), suggesting some inconsistency between evaluators when considering absolute differences.

When subjectively grading the small intestinal diameter to L5 vertebral height, both reviewers predominantly scored “approximately 1× the height of L5” pre‐ and post‐maropitant citrate administration. One reviewer scored a mild increase in diameter in three dogs (1–1.5× the height of L5), and the second reviewer scored a moderate increase in diameter in only 1 dog (>1.5× the height of L5) after maropitant citrate administration. The subjective small intestinal diameter‐to‐L5 vertebral height ratio demonstrated “fair” reliability pre‐administration (ICC = 0.461, *p *= 0.035) and “poor” reliability post‐administration (ICC = 0.098, *p *= 0.672). The rater difference ICC was similarly poor (ICC = 0.158, *p *= 0.494).

The average SI:L5 ratio prior to administration of maropitant citrate was 1.39 (range 1–1.87). The average ratio after administration was 1.36 (range 0.91–1.91). No significant difference was found between SI:L5 ratios before and after treatment (*p* = 0.553).

## 4 Discussion

After administration of maropitant citrate, the number of peristaltic contractions in the stomach was significantly decreased between 0 (baseline) and 4 h, with further significant decrease when patients had no peristalsis at baseline. Significant decrease in peristalsis in the jejunum after maropitant administration was not found when all patients were included but was found when patients with 0 contractions at baseline were excluded. These findings partially support the first hypothesis that maropitant citrate would decrease the rate of peristalsis of the gastrointestinal tract. The second hypothesis was not supported, as no statistically significant changes in radiographic small intestinal diameter, volume of gas content, or ultrasonographic luminal diameter were found. The pyloric, duodenal, and jejunal wall thickness was also not statistically changed posttreatment, other than the pyloric submucosal layer, which increased between the 0 and 1‐h time points. The underlying mechanism of this change is unknown, and it is likely not significant in a clinical setting.

Previous investigations have reported an average rate of peristalsis of 4–6 contractions per minute of the stomach and proximal duodenum, and 1–3 contractions per minute of the jejunum in normal dogs [17]. Multiple disease processes, environmental factors, and medications can cause gastrointestinal hypomotility and gastrointestinal signs such as vomiting [18, 19, 20, 21, 22, 23]. A major cause of these signs in dogs is small intestinal mechanical obstruction. With intestinal obstruction, the gastrointestinal tract initially becomes hyperperistaltic, causing fluid and gas accumulation in the small intestine orad to the obstruction and emptying of the aboral segment, leading to two populations of small intestinal diameter. Intestinal distention triggers the release of the neurotransmitter substance P, which binds to NK‐1 receptors in the gastrointestinal tract and stimulates secretion of electrolytes and water into the small intestinal lumen [3, 9]. The presence of two populations of bowel is commonly relied upon in the radiographic diagnosis of small intestinal mechanical obstruction. Similar changes are seen ultrasonographically in cases of small intestinal mechanical obstruction. A previous study examining radiographic and ultrasonographic findings in dogs with a suspected gastrointestinal foreign body reported the presence of fluid and/or gas distention of the orad gastrointestinal tract as common findings for both modalities [24]. The extent of small intestinal distention depends on several factors, including the degree of obstruction (full versus partial), the length of time the obstruction has been present, and the portion of small intestine obstructed (proximal duodenum vs. distal jejunum or ileum). Although there were no statistical differences in the intestinal size or volume of gas content in this normal dog population post maropitant administration, administration of maropitant prior to obtaining radiographic images in dogs with a small intestinal mechanical obstruction could affect the ability to diagnose a small intestinal mechanical obstruction due to the inhibition of actions of substance P at NK‐1 receptors. Further studies in dogs with small intestinal mechanical obstructions are needed.

When evaluating the number of contractions using all subjects, the only significant change was seen at the pylorus between the baseline and 1‐h time points. However, five of our subjects had zero baseline contractions at the pylorus, five at the duodenum, and 12 in the jejunum. Because this zero baseline contraction number makes it impossible to determine if maropitant citrate decreased motility, subjects with no baseline contractions were excluded. Statistical differences were then found, including decreased peristalsis at the pylorus (with a more significant decrease between baseline and 1‐h and additional significant decrease between baseline and 3‐h) and a significant decrease in jejunal peristalsis between baseline and 1‐h. Interestingly, although the number of duodenal contractions decreased between baseline and post‐maropitant citrate administration, the changes were not statistically significant, even after excluding patients with zero baseline contractions. One hypothesis for this is variability in the expression and affinity of NK‐1 receptors within segments of the gastrointestinal tract, though immunohistochemistry would be needed for investigation. Differences in the distribution of NK‐1 receptors throughout the canine gastrointestinal tract have not been reported, although differences in NK‐1 receptor expression have been reported with ileal inflammation in dogs [9].

The impacts of maropitant citrate on gastric peristalsis warrant additional consideration in any patient with this medication in their treatment plan. Reduced peristalsis may lead to delayed gastric emptying, leading to accumulation of ingesta and fluids in the stomach, increasing the risk of reflux or regurgitation and aspiration pneumonia. Certainly, in cases such as pancreatitis, where functional ileus is already of concern, careful consideration of the impacts of decreased gastric peristalsis is warranted.

The only difference in wall thickness measurements between time points was found in the pyloric submucosa, where a significant increase in thickness was found between baseline and 1 h. Interestingly, overall wall thickness was not significantly affected. The underlying mechanism for this change is unclear, as NK‐1 receptors are found within both the myenteric and submucosal plexuses, and a false positive finding (Type‐1 error) is possible [8]. Diffuse changes to wall layering thickness can occur in inflammatory bowel disease or be caused by infectious etiologies such as Heterobilharzia [25, 26]. These processes often present with gastrointestinal signs, and maropitant citrate is commonly part of their therapeutic plan. On the basis of these results, if diffuse changes to gastric or small intestinal wall layers are seen in these patients, this is likely not secondary to administration of maropitant.

Small intestinal diameter was assessed in both subjective and objective ways. These analyses were performed with two methods to ensure that significant differences were not overlooked when assessing the radiographs subjectively, without the use of calipers. Subjective assessment is commonly done when comparing pre‐ and post‐radiographic studies, especially in cases of reassessing the gastrointestinal tract for evidence of obstruction. No differences in small intestinal diameter were found using either method.

This study has multiple limitations. All dogs enrolled in this study were healthy, without apparent gastrointestinal signs. Although the authors suspect that these findings would persist in animals presenting with a gastrointestinal mechanical obstruction or other gastrointestinal disease, this is unknown, and further study is needed to examine the effects of maropitant citrate in a cohort of clinically affected dogs. All of the dogs were fasted for this study, which is known to cause a decreased rate of peristalsis; however, the authors do not expect that this had a significant impact on this study because each dog served as its own control, and we excluded dogs with zero baseline peristalsis. A fasted state was chosen to decrease variability in the effect of degree of stomach distention and timing of the last meal in an attempt to mitigate any possible short‐term effects of diet type on motility, to aid in ultrasonographic identification of the pylorus, and to better visualize intestinal segments and layering. The authors also believe that a fasted state more closely mimics a clinical scenario in which patients experiencing gastrointestinal signs would present with decreased gastric content due to vomiting, anorexia, and/or medically prescribed fasting. Lastly, some patients having zero baseline contractions led to analyzing the rate of peristalsis before and after their inclusion, which decreased the number of dogs included in each analysis. Excluding dogs with zero baseline contractions led to a smaller cohort, which was smaller than the initial sample number determined by power analysis and was less ideal for statistical analysis. Lastly, although maropitant citrate is reported to reach peak plasma concentrations within 1 h of subcutaneous administration, it is possible that the full effect on gastrointestinal motility would take longer than 3 h to fully manifest [6, 7].

This study brings to light a challenge in interpreting abdominal ultrasounds in patients presenting with signs of gastrointestinal disease who have also received maropitant citrate. If decreased gastrointestinal motility is noted, it would be clinically useful to know, but impossible to determine if ileus is secondary to primary pathology, secondary to maropitant citrate administration, or a combination thereof. Further studies on the effects of maropitant citrate on gastrointestinal motility in dogs presenting with suspected mechanical obstruction would be helpful. The authors hypothesize, based on the results of this preliminary study in healthy dogs, that maropitant citrate may prevent the development of two populations of small intestines and distention orad to a mechanical obstruction due to its inhibition of substance P at NK‐1 receptors, which could lead to delayed diagnosis and treatment in such cases.

In summary, this study found a significant decrease in the rate of peristalsis at the pylorus 1 h after maropitant citrate administration in healthy dogs. After excluding subjects with no peristaltic contractions during the initial baseline ultrasound prior to treatment with maropitant, there was also a significant decrease in the number of peristaltic contractions in the stomach 3 h after administration and in the jejunum 1 h after maropitant administration. Clinically significant changes to gastrointestinal wall thickness or layering were not found. No differences were found in radiographic small intestinal diameter or estimated volume of gas content. The authors recommend that administration of maropitant citrate be taken into consideration when interpreting abdominal radiographs and gastrointestinal ultrasound in dogs presenting with gastrointestinal symptoms, especially when there is concern for gastric stasis. Additional studies are needed to assess the effects of maropitant citrate in dogs with gastrointestinal disease and to determine if maropitant citrate may complicate the diagnosis of mechanical obstruction.

## Author Contributions

**Jillian Myers**: conceptualization, investigation, funding acquisition, writing – original draft, methodology, writing – review and editing. **Andra Voges**: conceptualization, investigation, funding acquisition, writing – review and editing. **Robert Werner**: conceptualization, writing – review and editing, supervision. **Nicola Ritter**: Analysis and interpretation of data.

## Funding

This study was funded by Texas A&M University College of Veterinary Medicine and Biomedical Sciences, Large Animal Clinical Sciences.

## Disclosure

Oral abstract was presented at the 2024 ACVR Annual Conference, Norfolk Virginia.

## Conflicts of Interest

The authors declare no conflicts of interest.

## Data Availability

Inquiries for supporting data may be directed to the corresponding author listed above.

## Endnotes

1 Zoetis Inc, Kalamazoo, MI, USA.

2 DFMTS, Del Medical, Bloomingdale, USA.

3 Samsung RS80A, Samsung RS85, Samsung Medical Co. LTD, Seoul, Republic of Korea.

4 Mach 7 Technologies, South Burlington, USA.

5 SPSS 18.0, SPSS Inc., Chicago, IL, USA.
